# Analysis of the Noise-Induced Regimes in Ricker Population Model with Allee Effect via Confidence Domains Technique

**DOI:** 10.1155/2014/346239

**Published:** 2014-05-28

**Authors:** Irina Bashkirtseva, Lev Ryashko

**Affiliations:** Ural Federal University, Lenina 51, Ekaterinburg 620083, Russia

## Abstract

We consider a discrete-time Ricker population model with the Allee effect under the random disturbances. It is shown that noise can cause various dynamic regimes, such as stable stochastic oscillations around the equilibrium, noise-induced extinction, and a stochastic trigger. For the parametric analysis of these regimes, we develop a method based on the investigation of the dispersions and arrangement of confidence domains. Using this method, we estimate threshold values of the noise generating such regimes.

## 1. Introduction

Environmental noise is an inevitable attribute of any living system. Investigations of noise-induced phenomena in biological systems attract the attention of many researchers [1–4]. Obviously, noise changes the quantitative properties of the system dynamics. Moreover, even small deterministic disturbances and stochastic fluctuations can cause abrupt catastrophic qualitative shifts in ecosystems [5–7]. Theoretically, such shifts can be attributed to the multistability of the corresponding nonlinear mathematical models. Due to nonlinearity, these dynamic models exhibit coexisting attractors, nonuniformity of phase portraits, and high sensitivity of boundaries of basins of attraction. Under the random disturbances, a phase trajectory can cross a separatrix between basins of the attraction of coexisting attractors and exhibit new dynamical regimes [5, 8]. In spatial population models, noise can generate pattern formations [9–11].

A classic example of noise-induced phenomena in ecosystems is an extinction of the population in the stochastic models with the Allee effect. The Allee effect means that there is a threshold population level below which the population goes to extinction. Deterministic population models with Allee effect are fairly well studied [12–18]. An analysis of stochastic population continuous-time models with the Allee effect is presented in [19–23].

In the present paper, we study Allee effect in the discrete-time population Ricker model forced by additive and parametric noises. For discrete-time systems, an exhaustive mathematical description of the stochastic dynamics in terms of probabilistic distributions requires a solution of the Frobenius-Perron equation [24, 25]. An analytical solution of this functional equation even for one-dimensional systems is possible only in very special cases. In these circumstances, for the description of stochastic attractors, a method of direct numerical simulation is widely used. This method requires a lot of computing power, so a development of the analytical approximations is a highly relevant area of research [26–28].

In the present paper, for the general one-dimensional discrete-time systems with parametric noise, we develop a new analytical method for the approximation of the dispersions of random states around stochastically forced equilibria. Mathematical background of this method is shortly presented in Section 2.

In Section 3, for the modified Ricker population model with Allee effect, we study probabilistic mechanisms of the noise-induced extinction and generation of stochastic trigger regime. Here, we demonstrate constructive abilities of the new approach based on confidence domains technique.

## 2. Analysis of Stochastic Equilibrium

Consider a deterministic system

(1) $$\begin{array}{c} x_{t+1}=f\left(x_{t}\right), \end{array}$$

where *f*(*x*) is a smooth scalar function. It is supposed that the system (1) has exponentially stable equilibrium $\overset{-}{x}$. It means that $f(\overset{-}{x})=\overset{-}{x}$ and $\left|f^{\prime }\left(\overset{-}{x}\right)\right|<1$.

Along with the deterministic system (1), consider a stochastically forced system

(2) $$\begin{array}{c} x_{t+1}=f\left(x_{t}\right)+\sigma \left(x_{t}\right)\xi_{t}, \end{array}$$

where *σ*(*x*) = (*σ*_1_(*x*),…, *σ*_*n*_(*x*)) is a smooth *n*-vector function and *ξ*_*t*_ = (*ξ*_1,*t*_,…, *ξ*_*n*,*t*_)^*⊤*^ is uncorrelated *n*-vector random process with parameters E*ξ*_*t*_ = 0, E(*ξ*_*t*_ *ξ*_*t*_^*⊤*^) = *V*, *t* = 0,1,…. Here, *n* × *n*-matrix *V* defines second moments of the coordinates of the vector *ξ*_*t*_. The function *σ*(*x*) describes a dependence of the intensity of random disturbances on the state of the system.

For the deviations $z_{t}=x_{t}-\overset{-}{x}$ of the system (2) states *x*_*t*_ from the equilibrium $\overset{-}{x}$, the following first approximation system holds:

(3) $$\begin{array}{c} z_{t+1}=f^{\prime }\left({\overset{-}{x}}_{t}\right)z_{t}+\left(\sigma \left(\overset{-}{x}\right)+\sigma^{\prime }\left(\overset{-}{x}\right)z_{t}\right)\xi_{t}, \end{array}$$

where *σ*′(*x*) = (*σ*_1_′(*x*),…, *σ*_*n*_′(*x*)). It follows from (3) that

(4) zt+12=[f′(x−)]2zt2+2f′(x−)[σ(x−)+σ′(x−)zt]ztξt+σ(x−)ξtξt⊤σ⊤(x−)+[σ(x−)ξtξt⊤σ′⊤(x−)+σ′(x−)ξtξt⊤σ⊤(x−)]zt+σ′(x−)ξtξt⊤σ′⊤(x−)zt2.

Consider the dynamics of the first two moments *m*_*t*_ = E*z*_*t*_, *D*_*t*_ = E*z*_*t*_^2^ for the system (3). From (3) and (4), due to the noncorrelatedness of *z*_*t*_ and *ξ*_*t*_, it follows that

(5) mt+1=amt,Dt+1=bDt+μmt+α,

where

(6) a=f′(x−),  b=a2+β,      α=σ(x−)Vσ⊤(x−),  β=σ′(x−)Vσ′⊤(x−),μ=σ(x−)Vσ′⊤(x−)+σ′(x−)Vσ⊤(x−).

Suppose that the nonlinear randomly forced system (2) has a stochastic attractor with the stationary stable probabilistic distribution. For the approximation of the two first moments of the random states of this probabilistic distribution near equilibrium $\overset{-}{x}$, we will use a stable stationary solution of the system (5).

Due to the condition of stability $\left|f^{\prime }\left(\overset{-}{x}\right)\right|<1$, for any initial value *m*_0_, the sequence *m*_*t*_ is stabilized:

(7) $$\begin{array}{c} \underset{t\to \infty }{\lim}m_{t}=0. \end{array}$$

A necessary and sufficient condition of the convergence of the sequence *D*_*t*_ to the constant value *M* is the inequality

(8) $$\begin{array}{c} b<1. \end{array}$$

Here,

(9) $$\begin{array}{c} M=\frac{\alpha }{1-b}=\frac{\sigma \left(\overset{-}{x}\right)V\sigma^{⊤}\left(\overset{-}{x}\right)}{1-{\left[f^{\prime }\left(\overset{-}{x}\right)\right]}^{2}-\sigma^{\prime }\left(\overset{-}{x}\right)V\sigma^{\prime ⊤}\left(\overset{-}{x}\right)}. \end{array}$$

**Remark 1**

If all noises in system (2) are additive, the functions *σ*_*i*_(*x*) are independent of *x* and, therefore, *β* = 0, $b={\left[f^{\prime }(\overset{-}{x})\right]}^{2}$, and condition (8) of the existence of stable stationary solutions of the system (5) is equivalent to the condition $\left|f^{\prime }\left(\overset{-}{x}\right)\right|<1$ of the stability of the equilibrium $\overset{-}{x}$. If the intensity of noise depends on the system (2) state (*β* > 0), then just the stability of the deterministic equilibrium is not sufficient. Here, condition (8) applies a restriction ${\left[f^{\prime }(\overset{-}{x})\right]}^{2}+\sigma^{\prime }(\overset{-}{x})V\sigma^{\prime ⊤}(\overset{-}{x})<1$ on the parameters $\sigma^{\prime }(\overset{-}{x})$ and *V* of noise. If this restriction is not satisfied, then the sequence of the second moments *D*_*t*_ given by the system (5) will increase indefinitely.

**Remark 2**

For the geometrical description of the scattering of random states, the confidence intervals are widely used. For scalar Gaussian random variable with mean value $\overset{-}{x}$ and dispersion *D*, the confidence interval is $\left(\overset{-}{x}-r,\overset{-}{x}+r\right)$, where

(10) $$\begin{array}{c} r=c\sqrt{2D},\;\;c=\text{er}{\text{f}}^{-1}\left(P\right),\;\\ \;\;\mathrm{erf}\left(x\right)=\frac{2}{\sqrt{\pi }}\overset{x}{\underset{0}{\int }}e^{-t^{2}}dt, \end{array}$$

and *P* is a fiducial probability. Stationary distributed random states of the system (2) are localized in the neighborhood of the equilibrium $\overset{-}{x}$. Using dispersion value *M*, one can construct a confidence interval $\left(\overset{-}{x}-r,\overset{-}{x}+r\right)$ where $r=c\sqrt{2M}$.

**Remark**

Consider a stochastic system

(11) $$\begin{array}{c} x_{t+1}=f\left(x_{t}\right)+\sigma_{1}\left(x_{t}\right)\xi_{1,t}+\sigma_{2}\xi_{2,t} \end{array}$$

forced by only two noises: parametric noise with intensity *σ*_1_(*x*) and additive noise with intensity *σ*_2_. Here, *ξ*_1,*t*_ and *ξ*_2,*t*_ are uncorrelated scalar random processes with parameters

(12) $$\begin{array}{c} \text{E}\left(\xi_{i,t}\right)=0,\;\text{E}\left(\xi_{i,t}^{2}\right)=1\;\left(i=1,2\right);\;\;\;\;\\ \text{E}\left(\xi_{1,t}\xi_{2,t}\right)=0\;\left(t=1,2,\ldots \right). \end{array}$$

This system is a particular case of the system (2) with

(13) $$\begin{array}{c} \sigma \left(x\right)=\left(\sigma_{1}\left(x\right),\sigma_{2}\right),\;\;\xi_{t}=\left[\begin{array}{c} \xi_{1,t}\\ \xi_{2,t} \end{array}\right],\;\;\;\\ V=\left[\begin{array}{cc} 1 & \;\;0\\ 0 & \;\;1 \end{array}\right],\\ \;\;\alpha =\sigma_{1}^{2}\left(\overset{-}{x}\right)+\sigma_{2}^{2},\;\;\beta ={\left[\sigma_{1}^{\prime }\left(\overset{-}{x}\right)\right]}^{2},\;\;\\ b={\left[f^{\prime }(\overset{-}{x})\right]}^{2}+{\left[\sigma_{1}^{\prime }(\overset{-}{x})\right]}^{2}. \end{array}$$

It follows from (9) that, for this case,

(14) $$\begin{array}{c} M=\frac{\sigma_{1}^{2}\left(\overset{-}{x}\right)+\sigma_{2}^{2}}{1-{\left[f^{\prime }\left(\overset{-}{x}\right)\right]}^{2}-{\left[\sigma_{1}^{\prime }\left(\overset{-}{x}\right)\right]}^{2}}. \end{array}$$

In the next section, we apply this theory to the study of noise-induced phenomena in Ricker model with Allee effect.

## 3. Analysis of Stochastic Ricker Model with Allee Effect

Consider a general one-dimensional population model governed by the following discrete-time equation:

(15) $$\begin{array}{c} N_{t+1}=g\left(N_{t}\right)N_{t}, \end{array}$$

where *N* is a size of the population and *g*(*N*) is a per capita intrinsic growth rate function. Value *N* = 0 is a trivial equilibrium of this system.

The dynamics of the system (15) is defined by the features of the function *g*(*N*). The condition *g* > 1 implies a growth of the population size; for *g* < 1, the population decreases. Solutions of the equation *g*(*N*) = 0 define the other equilibria of (15).

**Figure 1:**
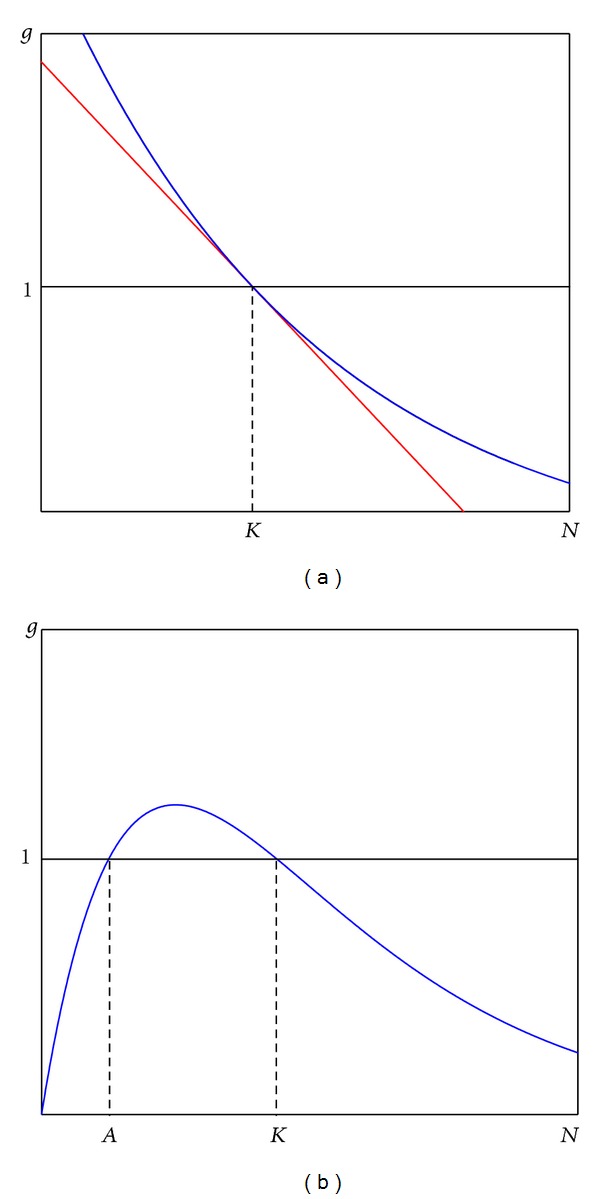
Intrinsic growth rate function *g*(*N*): (a) without Allee effect; (b) with Allee effect.

The simplest examples of the functions *g*(*N*) are plotted in Figure 1(a). As one can see, for sufficiently small values *N*_0_, due to *g*(*N*_0_) > 1, the population grows. On the contrary, for large *N*_0_, the population decreases. The value *N* = *K* is a nontrivial equilibrium of the system (15). If this equilibrium is unstable, then the system (15) can exhibit different types of dynamics with periodic or chaotic oscillations around the equilibrium *N* = *K*.

This type of dynamics is observed for well-known discrete-time models such as Verhulst equation with *g*(*N*) = 1 + *r*(1 − (*N*/*K*)) (red line in Figure 1(a)) and Ricker system with *g*(*N*) = exp⁡[*r*(1 − (*N*/*K*))] (blue line in Figure 1(a)). Such type of the function *g*(*N*) considered here adequately reflects a survival law: the more the population size, the less the growth rate.

For small size of the population, the function *g*(*N*) has to describe a birth rate mainly. But there are many ecological situations when at a low population level the function *g*(*N*) is significantly less than unit, and *g*(*N*) vanishes as the size of the population tends to zero. If we assume that the birth rate is proportional to the population size as the population level is low, one gets another type of the function *g*(*N*) (see Figure 1(b)).

As one can see, this new function now provides another type of the population dynamics for small values of *N*_0_. Indeed, one more equilibrium *N* = *A* for (15) appears. If *N*_0_ < *A*, then the sequence *N*_*t*_ monotonically tends to zero. This means an extinction of the population. If *N*_0_ is slightly more than *A*, then the population grows and its future behavior is defined by features of the function *g*(*N*) for *N* > *A*. So, the value *N* = *A* is a threshold value separating two different types of population dynamics. Such a phenomenon of the existence of a threshold population level, below which the population goes to extinction, is called an Allee effect [12, 16].

To incorporate an Allee effect into the classical Ricker model, we use the following modification [29] of the growth rate function:

(16) $$\begin{array}{c} g\left(N\right)=\frac{N}{A}\exp\left[r\left(1-\frac{N}{A}\right)\right]. \end{array}$$

For this function, due to *g*(*A*) = 1, (15) has an equilibrium *N* = *A* corresponding to the Allee threshold. Another nontrivial equilibrium *N* = *K* can be found from the following equation:

(17) $$\begin{array}{c} K\exp\left[r\left(1-\frac{K}{A}\right)\right]=A. \end{array}$$

For dimensionless new variable *x* = *N*/*A*, (15) can be rewritten as

(18) $$\begin{array}{c} x_{t+1}=x_{t}\varphi \left(x_{t}\right)=f\left(x_{t}\right),\;\;\varphi \left(x\right)=x\exp\left[r\left(1-x\right)\right]. \end{array}$$

For the zone 0 < *r* < 1, the system (18) has three equilibria: ${\overset{-}{x}}_{0}=0$, ${\overset{-}{x}}_{1}=1$, and ${\overset{-}{x}}_{2}(r)>{\overset{-}{x}}_{1}$. The equilibrium ${\overset{-}{x}}_{0}$ is stable, the equilibrium ${\overset{-}{x}}_{1}$ is unstable, and the equilibrium ${\overset{-}{x}}_{2}(r)$ is stable for 0.1788 < *r* < 1. For *r* < 0.1788, the system performs a Feigenbaum scenario of period-doubling bifurcations and transition to chaos. The unstable equilibrium ${\overset{-}{x}}_{1}$ separates basins of attraction of the stable equilibrium ${\overset{-}{x}}_{0}$ and attractors (both regular and chaotic) arranged in the zone $x>{\overset{-}{x}}_{1}$. Here, ${\overset{-}{x}}_{1}$ plays a role of the threshold population level of Allee effect: if the initial density *x*_0_ of the population is below ${\overset{-}{x}}_{1}$, then it goes to the extinction: lim⁡_*t*→*∞*_ *x*_*t*_ = 0.

**Figure 2:**
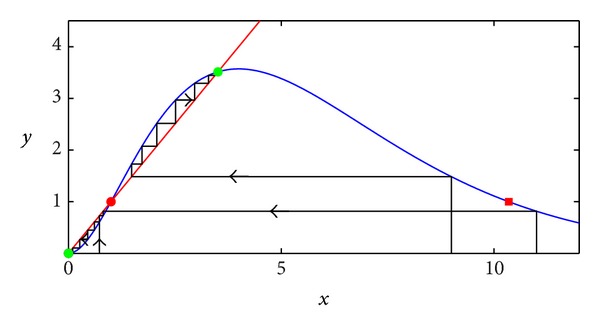
Dynamics of the deterministic Ricker model (18) with Allee effect for *r* = 0.5.

In what follows, we focus on the case when ${\overset{-}{x}}_{2}$ is stable and fix *r* = 0.5. In Figure 2, for *r* = 0.5, the dynamics of the system (18) for the different initial values *x*_0_ is shown. Here, stable equilibria ${\overset{-}{x}}_{0}=0$ and ${\overset{-}{x}}_{2}=3.513$ are plotted by green circles; the unstable equilibrium ${\overset{-}{x}}_{1}=1$ is plotted by red circle. A red square marks a right border of the interval of attraction of the equilibrium ${\overset{-}{x}}_{2}$.

For the Ricker model in this deterministic case, there are only two variants of the dynamics: the population goes either to extinction or to the positive equilibrium. Such a strong separation of dynamical regimes can be destroyed by a noisy environment.

Along with the deterministic model (18), we will consider the stochastic system

(19) xt+1=xt[φ(xt)+σ1ξ1,t]+σ2ξ2,t=xt2exp⁡[r(1−xt)]+σ1xtξ1,t+σ2ξ2,t,

where *ξ*_1,*t*_ and *ξ*_2,*t*_ are uncorrelated scalar random processes with parameters

(20) $$\begin{array}{c} \text{E}\left(\xi_{i,t}\right)=0,\;\text{E}\left(\xi_{i,t}^{2}\right)=1\;\left(i=1,2\right);\;\;\;\;\\ \text{E}\left(\xi_{1,t}\xi_{2,t}\right)=0. \end{array}$$

Here, *σ*_1_ is an intensity of the parametric noise modeling the disturbances of the rate function *φ*(*x*), and *σ*_2_ is an intensity of the external additive noise.

Under stochastic disturbances, the solutions of (19) leave the deterministic equilibria and form some stationary probabilistic distributions around points ${\overset{-}{x}}_{0}$ and ${\overset{-}{x}}_{2}$. For the analysis of the dispersions of the random states, the approximations (14) are used. For dispersions in the system (19), around the equilibrium ${\overset{-}{x}}_{0}$ we have

(21) $$\begin{array}{c} M_{0}=\frac{\sigma_{2}^{2}}{1-\sigma_{1}^{2}}, \end{array}$$

and around the equilibrium ${\overset{-}{x}}_{2}$ we have

(22) $$\begin{array}{c} M_{2}=\frac{\sigma_{1}^{2}{\overset{-}{x}}_{2}^{2}+\sigma_{2}^{2}}{1-{\left(f^{\prime }\left({\overset{-}{x}}_{2}\right)\right)}^{2}-\sigma_{1}^{2}}. \end{array}$$

When the noise intensity is quite small, random states leaving a stable deterministic equilibrium are concentrated around it with the small dispersion. As the noise intensity increases, a dispersion of random states increases too, and the system (19) can exhibit qualitative changes of stochastic dynamics.

Upon reaching a certain critical value of the noise intensity, iterations of the stochastic system (19) with a high probability pass through the unstable equilibrium ${\overset{-}{x}}_{1}$ into the basin of attraction of the stable equilibrium ${\overset{-}{x}}_{0}$ and perform small-amplitude stochastic oscillations near ${\overset{-}{x}}_{0}$. Biologically, this phenomenon can be interpreted as a noise-induced extinction of the population. Evaluation of the critical noise intensity corresponding to the beginning of these transitions may be obtained on the base of confidence domains technique. Functions (21) and (22) give us explicit parametrical description for confidence intervals $I_{0}=({\overset{-}{x}}_{0}-r_{0},{\overset{-}{x}}_{0}+r_{0})\;\;$ and $I_{2}=({\overset{-}{x}}_{2}-r_{2},{\overset{-}{x}}_{2}+r_{2})$, where $r_{i}=\text{er}{\text{f}}^{-1}(P)\sqrt{2M_{i}}$.

**Figure 3:**
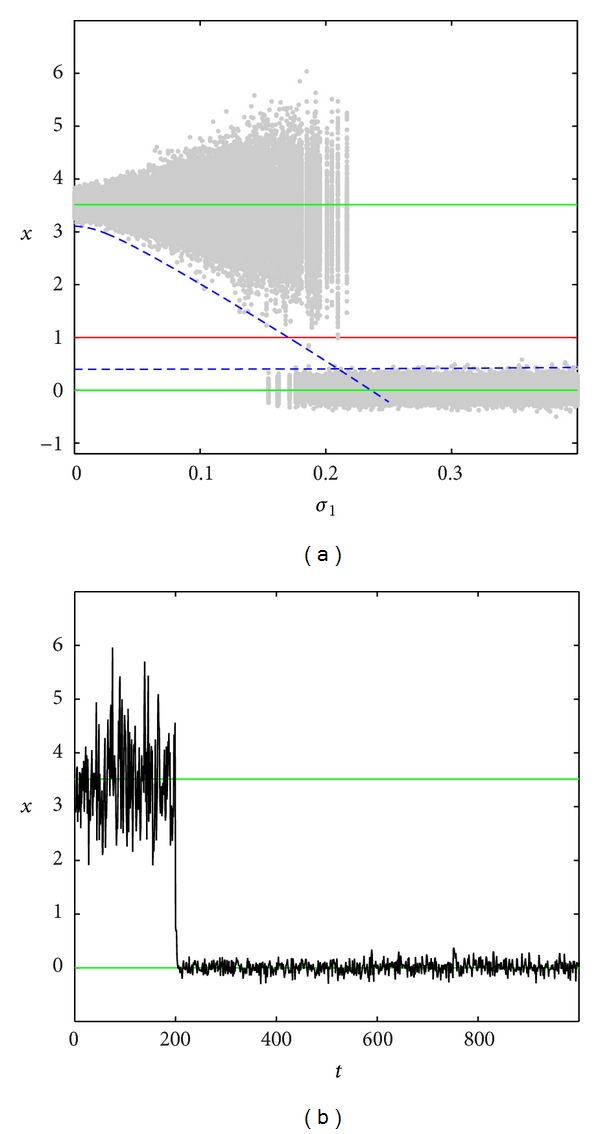
Noise-induced transitions for *σ*_2_ = 0.1.

**Figure 4:**
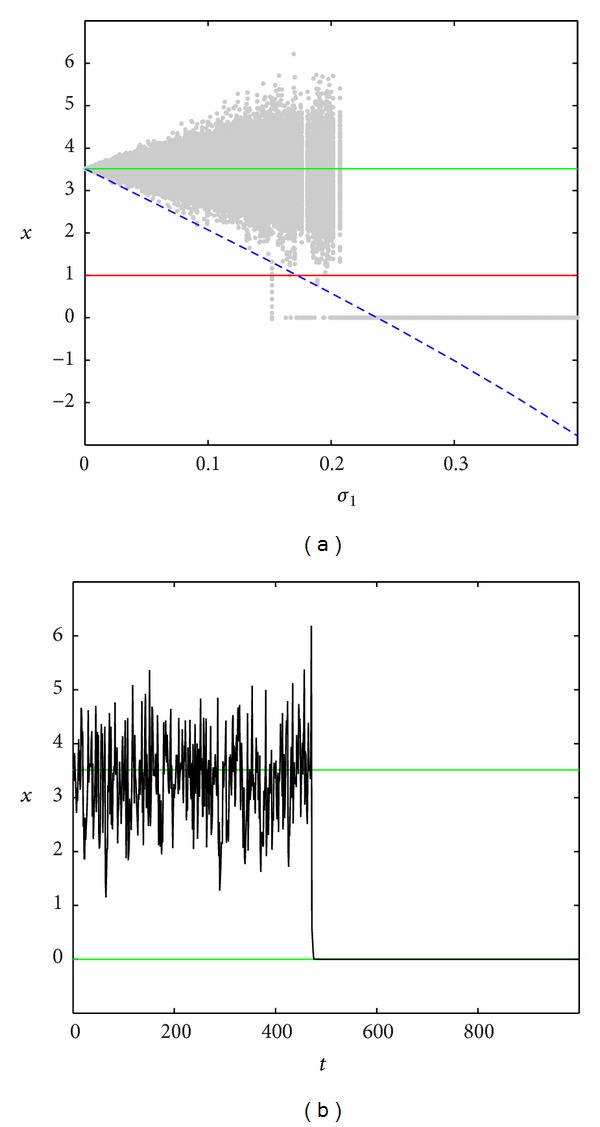
Noise-induced transitions for *σ*_2_ = 0.

**Figure 5:**
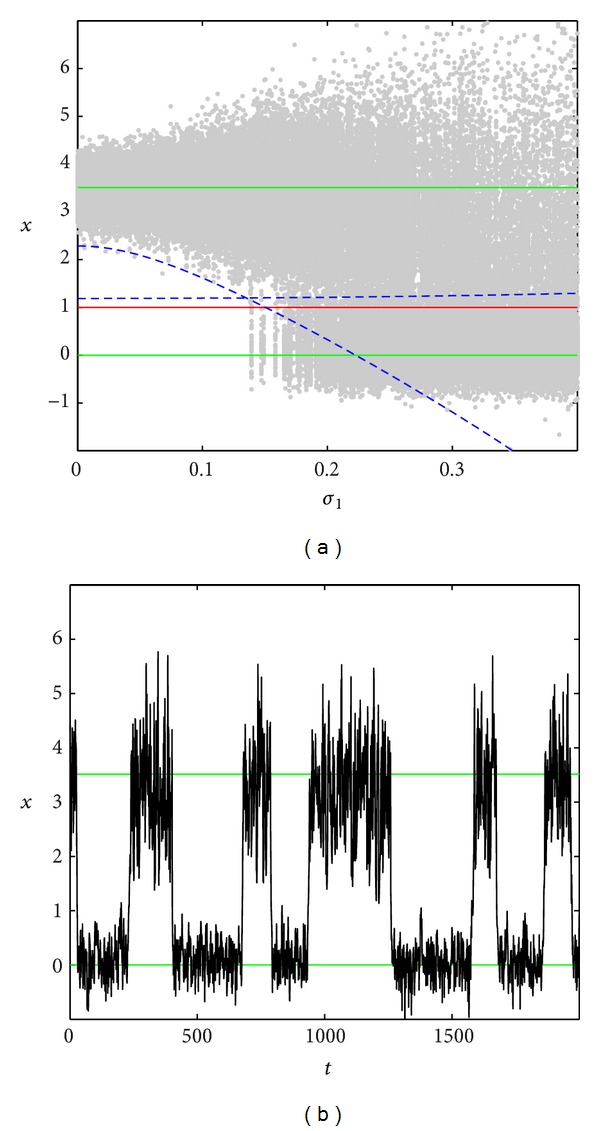
Noise-induced transitions for *σ*_2_ = 0.3.

In Figures 3–5, the dependence of the stochastic attractors on the intensity *σ*_1_ of parametric noise for the fixed additive noise intensity *σ*_2_ is presented. Here, random states (grey color) of attractors have been found by the direct numerical simulation. The stable equilibria ${\overset{-}{x}}_{0}$, ${\overset{-}{x}}_{2}$ of the deterministic system are plotted by green lines, the unstable equilibrium ${\overset{-}{x}}_{1}$ is plotted by red line, and the nearest borders $x={\overset{-}{x}}_{0}+r_{0}(\sigma_{1})$ and $x={\overset{-}{x}}_{2}-r_{2}(\sigma_{1})$ of confidence intervals *I*_0_ and *I*_2_ are plotted by dashed blue lines. Here, the representative time series are also shown.

As one can see in Figure 3(a) for the fixed *σ*_2_ = 0.1, when *σ*_1_ exceeds threshold value *σ*_1_* ≈ 0.2, random trajectories with high probability demonstrate noise-induced transitions from the basin of attraction of the nontrivial equilibrium ${\overset{-}{x}}_{2}$ to the small vicinity of the equilibrium ${\overset{-}{x}}_{0}$.

An estimation of the critical value of *σ*_1_* can be derived from the mutual arrangement of the separatrix ${\overset{-}{x}}_{1}$ and confidence intervals borders. In our case, the noise intensity that corresponds to the intersection of the lower border $x={\overset{-}{x}}_{2}-r_{2}(\sigma_{1})$ of the confidence interval *I*_2_ with the separatrix $x={\overset{-}{x}}_{1}$ can be used as an estimation of the threshold value *σ*_1_* ≈ 0.17. Here, the fiducial probability is *P* = 0.999.

As one can see, the value *σ*_1_* quite accurately localizes a qualitative change in the stochastic dynamics of the studied system. Note that, after transition to the neighborhood of the trivial equilibrium, a dispersion of random states is well described by the confidence interval found for ${\overset{-}{x}}_{0}=0$. In Figure 3(b), a solution of the system (19) for *σ*_1_ = 0.2, *σ*_2_ = 0.1 illustrates the transition from the neighborhood of ${\overset{-}{x}}_{2}$ to the neighborhood of ${\overset{-}{x}}_{0}$.

Similar results can be obtained for the case when the system (19) is forced by parametric perturbations only (*σ*_1_ ≠ 0,  *σ*_2_ = 0). In this case (see Figure 4), solutions of (19) with high probability cross the separatrix ${\overset{-}{x}}_{1}=1$ and quickly vanish. Biological interpretation of this transition is a noise-induced extinction of the population.

As noise grows, both confidence intervals around the ${\overset{-}{x}}_{0}$ and ${\overset{-}{x}}_{2}$ expand beyond the boundary $x={\overset{-}{x}}_{1}$ of the basins of attraction of these stable equilibria and begin to overlap each other (see Figure 5(a) for *σ*_2_ = 0.3). As a consequence, the system exhibits repeated random transitions between these basins.  Figure 5(b) for *σ*_1_ = 0.2, *σ*_2_ = 0.3 demonstrates two types of stochastic oscillations: oscillations near equilibria and noise-induced transitions between the neighborhoods of equilibria. In this case, the population can be characterized as a stochastic trigger.

So, in the presence of noise, the Ricker population model with Allee effect represents various types of stochastic dynamics: long-term random oscillations with the small dispersion near the positive equilibrium, noise-induced extinction, and stochastically generated trigger. The new mathematical technique presented here provides a useful tool for the analytical estimation of the noise intensities corresponding to these regimes.

## 4. Conclusion

In this paper, we propose a new approach for the constructive study of noise-induced phenomena in the Ricker population model with the Allee effect. Theoretical basis of this approach is an analysis of dispersions of random states near deterministic equilibria of this bistable model. Using this theory, we construct confidence intervals around the stable equilibria. The mutual arrangement of these intervals and Allee threshold are used in the geometrical analysis of the various stochastic regimes in this model: stable stochastic oscillations around nontrivial equilibrium, noise-induced extinction, and stochastic trigger. Note that the elaborated method is readily applicable to more complicated models of multiple interacting populations forced by parametric noises.
